# Biomimetic synthesis of iron oxide nanoparticles from *Bacillus megaterium* to be used in hyperthermia therapy

**DOI:** 10.1186/s13568-022-01490-y

**Published:** 2022-11-19

**Authors:** Sajedeh Hajiali, Sara Daneshjou, Somayeh Daneshjoo

**Affiliations:** 1 Department of Nanobiomimetic, Faculty of Advanced Sciences and Technology, Tehran Medical Sciences, Islamic Azad University, Tehran, Iran; 2 Department of Nanobiotechnology, Faculty of Biological Science, Tarbiat Modares University, Tehran, Iran; 3 Department of Medical Nanotechnology, Faculty of Advanced Sciences and Technology, Tehran Medical Sciences, Islamic Azad University, Tehran, Iran

**Keywords:** *Bacillus megaterium*, Iron oxide, Nano-biomimetic, Hyperthermia

## Abstract

The suitable structural characteristics of magnetic nanoparticles have resulted in their widespread use in magnetic hyperthermia therapy. Moreover, they are considered a proper and operational choice for pharmaceutical nanocarriers. Using the biomimetic method, we were able to produce iron oxide magnetic nanoparticles from the bacterial source of PTCC1250, *Bacillus megaterium*, for therangostic diagnosis systems and targeted drug delivery. Some of the benefits of this method include mitigated environmental and biological dangers, low toxicity, high biocompatibility, cheap and short-term mass production possibilities in each synthesis round compared to other biological sources, simple equipment required for the synthesis; and the possibility of industrial-scale production. *Bacillus megaterium* is a magnetotactic bacteria (MTB) that has a magnetosome organelle capable of orienting based on external magnetic fields, caused by the mineralization of magnetic nanocrystals. Utilizing this capability and adding an iron nitrate solution to the bacterial suspension, we synthesized iron oxide nanoparticles. The extent of synthesis was measured using UV–visible spectrophotometry. The morphology was evaluated using FESEM. The crystallized structure was characterized using RAMAN and XRD. The size and distribution of the nanoparticles were assessed using DLS. The surface charge of the nanoparticles was measured using zeta potential. The synthesis of iron oxide nanoparticles was confirmed using FT-IR, and the magnetic property was measured using VSM. This study is continued to identify industrial and clinical applications.

## Keypoints

- Synthesis of iron nanoparticles for simultaneous diagnostic and therapeutic applications
- Production of magnetic iron oxide nanoparticles by magnetotactic bacteria with uniform size
- Using the synthesis pattern in Bacillus megatrium to produce nano-field iron oxide

## Introduction

The production and synthesis of magnetic nanoparticles in recent years due to their widespread applications have led to the focus of researchers all around the world on the design of suitable synthesis pathways to reach better performance and a higher level of applicability. Various issues, including inherent magnetic properties, the size and shape of the nanoparticles, stability in aquatic environments, non-toxicity of the surface coating, and the surface charge of the nanoparticles, must be considered when synthesizing magnetic nanoparticles. To do so, selecting a suitable synthesis method is essential for reaching the desired size and shape, suitable colloidal stability, and proper surface coating of the magnetic nanoparticles (McGuire et al. 2016). In this context, methods based on natural inspirations have resulted in producing materials with unique characteristics. As a bonus, such methods do not suffer from the disadvantages plaguing physicochemical methods, including high production costs, accumulation of toxic reagents, and constraints related to using such nanoparticles in biological applications. Consequently, the synthesis of nanoparticles using biological and nature-inspired methods can help improve our capability for producing desired nanoparticles for medicinal and pharmaceutical purposes (Wang and Wang 2014; Rajakumar et al. 2021; Turakhia et al. 2019; Saif et al. 2016). Examples of such studies can be seen in the green synthesis of iron from sources such as Indian lilac (Azadirachta indica), ginger roots, rambutan skin, bacteria, fungi, algae, and plants for the cheap and low-energy production of metal nanoparticles that are environmentally friendly (Zambri et al. 2019; Yuvakkumar and Hong 2014; Arakha et al. 2015; Xie et al. 2009; Xiao et al. 2020). Among the various methods proposed for nanoparticle biosynthesis, the use of bacteria is of particular importance (Hedayatnasab et al. 2020; Konishi 2004). Some of the benefits of such a production method include an abundant and easily-available source that provides quick returns, the high volume of nanoparticles produced in each round of synthesis, and compatibility with the environment. Metal nanoparticles have attracted much attention due to their unique optical characteristics, catalytic properties, and electrical and magnetic applications (Ankamwar et al. 2005). The use of iron oxide nanoparticles in pharmaceutical and drug-related research is based on their suitable magnetic properties, low toxicity, high compatibility, and easier synthesis compared to other nanoparticles (Colombo et al. 2012). Using microorganisms for synthesizing nanoparticles is gaining more popularity as an alternative to industrial methods to improve the characteristics of the synthesized nanoparticles. Magnetic hyperthermia therapy for cancer treatment is becoming more common as a suitable alternative to chemotherapy and radiotherapy due to its high targeting ability and low systemic toxicity (Simeonidis et al. 2020). Furthermore, using iron oxide magnetic nanoparticles in shell-core structures to improve the hyperthermia process for the diagnosis and/or treatment of diseases such as cancer has boosted the efficiency of hyperthermia through manipulating the interactions between the shell and core phases, resulting in changes in the specific absorption rate, the occurrence of reversed magnetic states affecting inter-particle interactions, and finally an increase in the conversion rate of energy into heat (Soleymani et al. 2020). Moreover, the single-vase hydrothermal method has been used for producing iron oxide nanoparticles coated with hyaluronic acid to create environmental compatibility and the identifier ligand for breast cancer cells. Due to its narrow surface charge distribution and colloidal stability under physiological conditions, environmental compatibility, and the property of targeting cancer cells, this product has several theranostic and biomedical applications (Dutta et al. 2020). Using iron oxide nanoparticles with a surface charge of − 31 mV with a high loading tendency toward the hydrophobic anti-breast cancer drug, curcumin, and the pH-response method, researchers have been able to study and develop applications for stabilized micelles of iron oxide nanoparticles for hyperthermia and to deliver the hydrophobic anti-cancer drugs (Andrade et al. 2020). So far, there have been many studies on iron oxide-based magnetic nanoparticles and their clinical applications. In addition, various attempts have been made to develop artificial and green synthesis pathways, resulting in different anisotropic shapes of magnetite (Fe_3_O_4_) nanoparticles and other ferrites (FeO) and the spherical shapes synthesized using common methods. It has been shown that controlling the shape of nanoparticles can improve magnetic behavior, biological activity and various properties such as surface and magnetic resonance imaging (MRI) and make them an excellent choice for medical applications (Yew et al. 2020). As a result, this study is done to synthesize iron oxide nanoparticles using *Bacillus megaterium* to obtain a biological nanocarrier with desirable magnetic properties and higher compatibility than other biological models synthesized for use in novel therapeutic applications including hyperthermia in the simultaneous diagnosis and treatment of diseases such as cancer.

## Materials and methods

### Materials and preparing the culture medium and the bacteria

This study used the nutrient broth microbial culture media (LB Broth, Merck KGaA, Germany). Moreover, the standard bacterial strain produced in the Center for the Collection of Industrial and Infectious Fungi and Bacteria of the Iranian Organization for Scientific and Industrial Research was utilized (*Bacillus megaterium* PTCC1250). In addition, iron (II) sulfate salt (Merck KGaA, Germany) was used for preparing the saline solution.

### Synthesizing the iron nanoparticles using Bacillus megaterium

To synthesize the iron nanoparticles from the biological source, *Bacillus megaterium*, the bacteria were cultured in the Nutrient Broth medium, followed by incubation at 37 ℃. After ensuring the growth of the bacteria, the bacterial suspension was mixed with the solution of iron sulfate at a concentration of 0.1 M with a ratio of 1:1, and the synthesis was performed at room temperature.

### UV–visible spectrophotometry (UV–vis)

The suspension containing the bacteria and the magnetic iron nanoparticles, after filtration, underwent UV–visible spectrophotometry. The absorption spectra of the synthesized nanoparticles were assessed using a spectrophotometric device (PerkinElmer Inc., Waltham, MA, USA) in the wavelength range of 200–700 nm at different timeframes.

### Imaging using field-emission scanning electron microscopy (FESEM)

A sufficient suspension containing the nanoparticles after filtration was transferred to a microscope slide using a capillary tube. After drying, the nanoparticles were imaged using the Sigma VP FESEM device (Zeiss, Germany), and size measurements were performed at 100 and 200 nm scales.

### Energy-dispersive X-ray spectroscopy (EDS)

Along with looking at the sample’s image and morphology, the sample's two-dimensional element map was obtained using EDS as a secondary technique qualitatively and quantitatively for the spatial distribution of the elements at the surface of the sample.

### X-ray diffraction (XRD)

To perform XRD analysis, the solution containing the synthesized nanoparticles was centrifuged three times at 12,000 rpm for 20 min at 24 ℃. The upper solution was then discarded, while the resulting residue was placed in an oven at 50 ℃ for 24 h to dry up. Finally, the dried powder of iron nanoparticles was assessed using an X Pert Pro XRD device (Panalytical) to evaluate the crystal structure of the nanoparticles.

### Fourier-transform infrared spectroscopy (FTIR)

After manufacturing caviar tablets using a capillary tube, a sufficient volume of the filtered sample suspension was transferred onto the tablets. After drying up, infrared spectroscopy was performed using a Nicolet IR100 FT-IR (Thermo Scientific) with the wavelength range of 300 to 4000 cm^–1^. Then, the vibration peak was compared to the standard peak for the vibration of $FeO$ to analyze the synthesis qualitatively.

### Dynamic light scattering (DLS) and zeta potential measurement

Dynamic light scattering (DLS) and the size of the particles were assessed by measuring the Brownian motion of the particles or the movement of the nanoparticles present in the suspension using a Zetasizer Zeta-DLS device (Malvern, UK). Moreover, the analysis of the zeta potential of the nanoparticles was performed by the device as a measure for determining the stability of the sample while determining the size of the nanoparticles.

### Raman spectroscopy

Raman spectroscopy was performed on the liquid samples using an XloRA Plus device (Horiba, Japan).

### Vibrating sample magnetometer (VSM)

Powdered samples were analyzed at room temperature using a vibrating sample magnetometer (VSM) manufactured by Daghigh Kavir Engineering Company (Iran). Furthermore, the values of magnetic parameters, including saturated magnetism, remaining magnetism, and coercive force were measured.

## Results

**Fig. 1:**
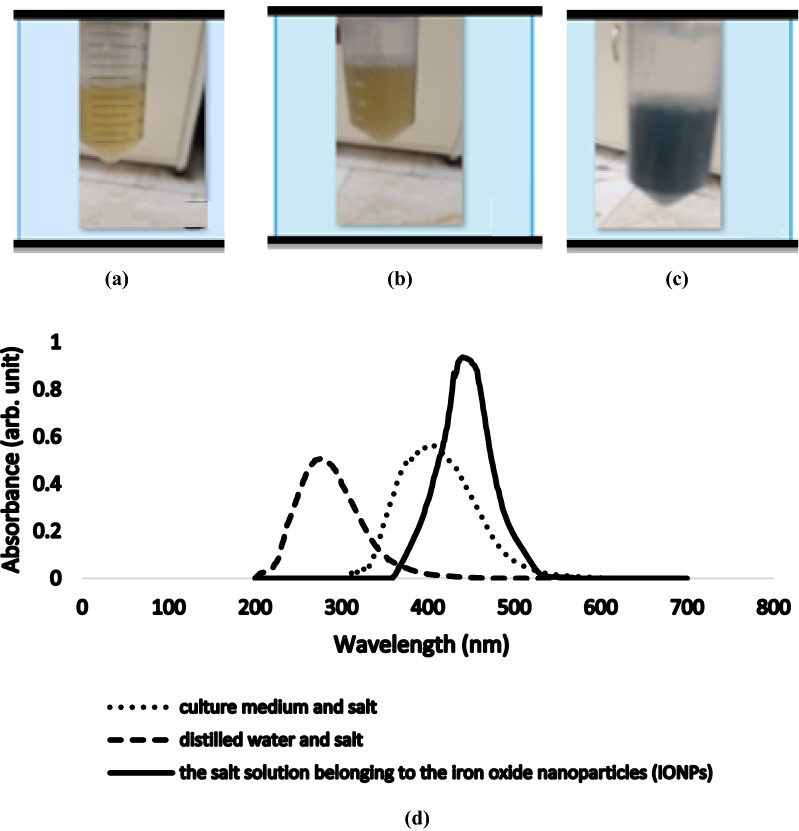
**a** Bacterial suspension, **b** bacterial suspension and the salt solution at the onset, **c** the bacterial suspension and the salt solution after 20 min, **d** UV–visible spectrophotometry (UV–vis)

After adding the salt solution to the bacterial suspension, a color shift from light yellow to orange and grayish-green over time was observed (Fig. 1a–c). Since a color shift is the first factor for confirming the synthesis of nanoparticles at the macroscopic scale, after observing this color shift, we performed more detailed analyses using spectrophotometry (Kebede et al. 2011; Koutzarova et al. 2006).

UV–visible spectrophotometry (UV–vis) was performed at a 200–700 nm wavelength on the suspension containing nanoparticles, distilled water, and 0.1 mM solution of iron nitrate as the first control or the Nutrient Broth culture medium and salt as the second control, respectively. A peak in the wavelength range of 250–320 nm corresponding to the first control, a peak in the wavelength range of 340–370 nm related to the second control, and a peak in the wavelength range of 370–450 nm associated with the bacterial suspension and the salt solution belonging to the iron oxide nanoparticles (IONPs) were observed. As can be seen in Fig. 1d, compared to the first control (distilled water and salt), the location of the peak is shifted toward longer wavelengths (i.e., redshift), while compared to the second control (culture medium and salt). We observed both a shift in the location of the peak toward a shorter wavelength and an increase in the height of the peak (i.e., hypsochromic shift). This indicates the establishment of a strong electrostatic attraction and a shift toward shorter wavelengths caused by the solvent effect, i.e., replacement, which confirms the synthesis of the iron oxide nanoparticles (Russ 2013; Chen et al. 2015). Moreover, we observed an increase in the height of the peak over time; but this trend did not change significantly after a week.

**Fig. 2:**
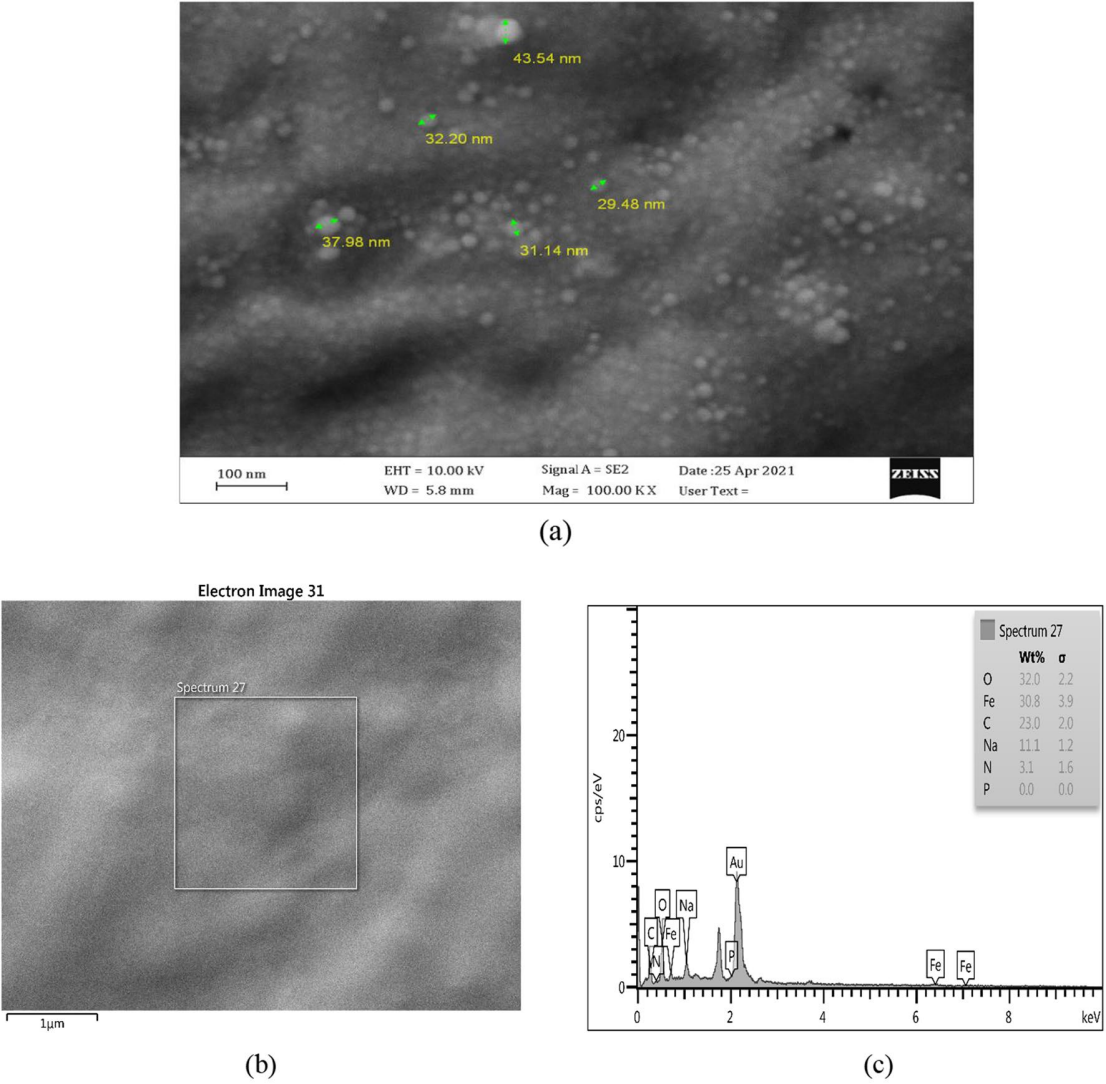
**a** Imaging using Field-Emission Scanning Electron Microscopy (FESEM), **b**, **c** element mapping using EDS

Imaging using Field-Emission Scanning Electron Microscopy (FESEM) at the scale of 100 nm indicated several particles in the range of 20–30 nm (Fig. 2a).

Along with observing the image and morphology of the sample using energy-dispersive X-ray spectroscopy (EDS), the elemental combination of the sample was qualitatively and quantitatively measured using Field-Emission Scanning Electron Microscopy (FESEM), indicating the presence of Fe and O elements (Fig. 2b, c). Moreover, according to the obtained results, the Fe element was present at low energy states and energy levels closer to the nucleus (Fe L $\alpha$) with a high concentration. At the same time, it was present with a low concentration at energy levels further from the nucleus at high energy states ($Fe \kappa \beta$). It should be noted that during the electron bombardment of the sample, the migration of the atoms from higher energy levels toward lower energy levels to reach a stable state will result in emitted photons. Therefore, the results of EDS indicated that the sample contained regular varieties of iron oxide (DeGaetano et al. 1992; Wang et al. 2021; Hashimoto et al. 2007; Mishra et al. 2014; Hashimoto et al. 2007; Devi et al. 2019a, b; Jagathesan and Rajiv 2018; Groiss et al. 2017; Devi et al. 2019a, b).

**Fig. 3:**
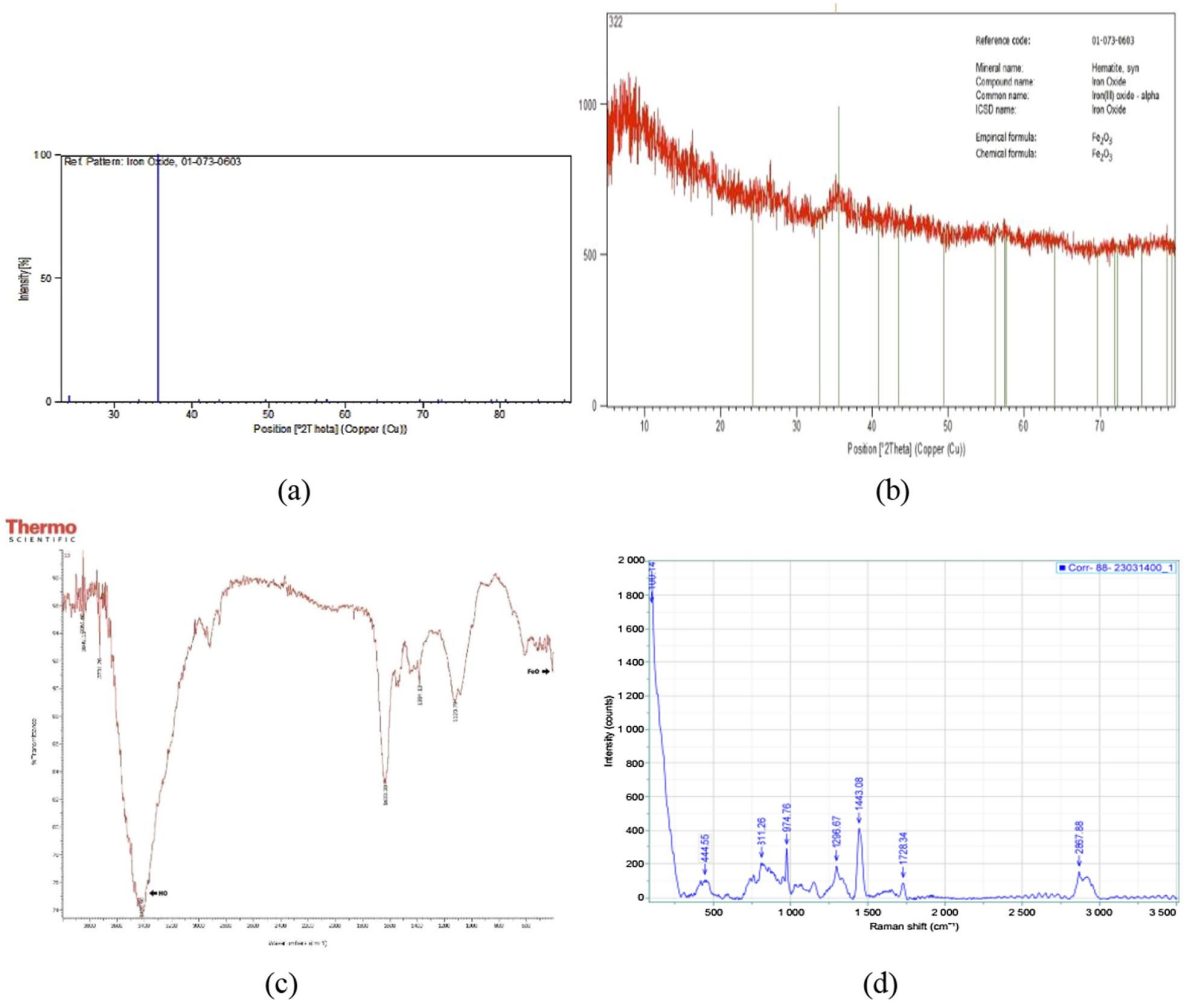
**a**, **b** X-ray diffraction (XRD) pattern for the suspension containing iron oxide, **c** Fourier-transform infrared spectroscopy (FTIR), **d** Raman spectroscopy

Performing X-ray diffraction (XRD) on the sample and matching the result with the standard diffraction sample of iron oxide-based on the source code of 01-073-0603 indicated the presence of sample 29, confirming the synthesis of iron nanoparticles. The peak observed for the sample at the 2^θ^ location was 35.2 with a height of 1.5 (Fig. 3a, b). Moreover, the results obtained from evaluating the crystallites and determining the phase and network parameters for the sample are presented in Table 1 (Saqib et al. 2019; Sharma and Jeevanandam 2013; Rajiv et al. 2017; Demirezen et al. 2019a, b; Fu 2015; Lim et al. 2013).

**Table 1:** Network parameters for the sample

Crystallographic parameters	
Crystal system	Hexagonal
Space group	R-3c
Space group number	167
a (A)	5.0342
b (A)	5.0342
c (A)	13.7483
Alpha	90.0000
Beta	90.0000
Gamma	120.0000
Calculated density (g/cm^3^)	5.27
Volume of cell (106 pm^3^)	301.75
z	6.00
RIR	0.06

**Fig. 4:**
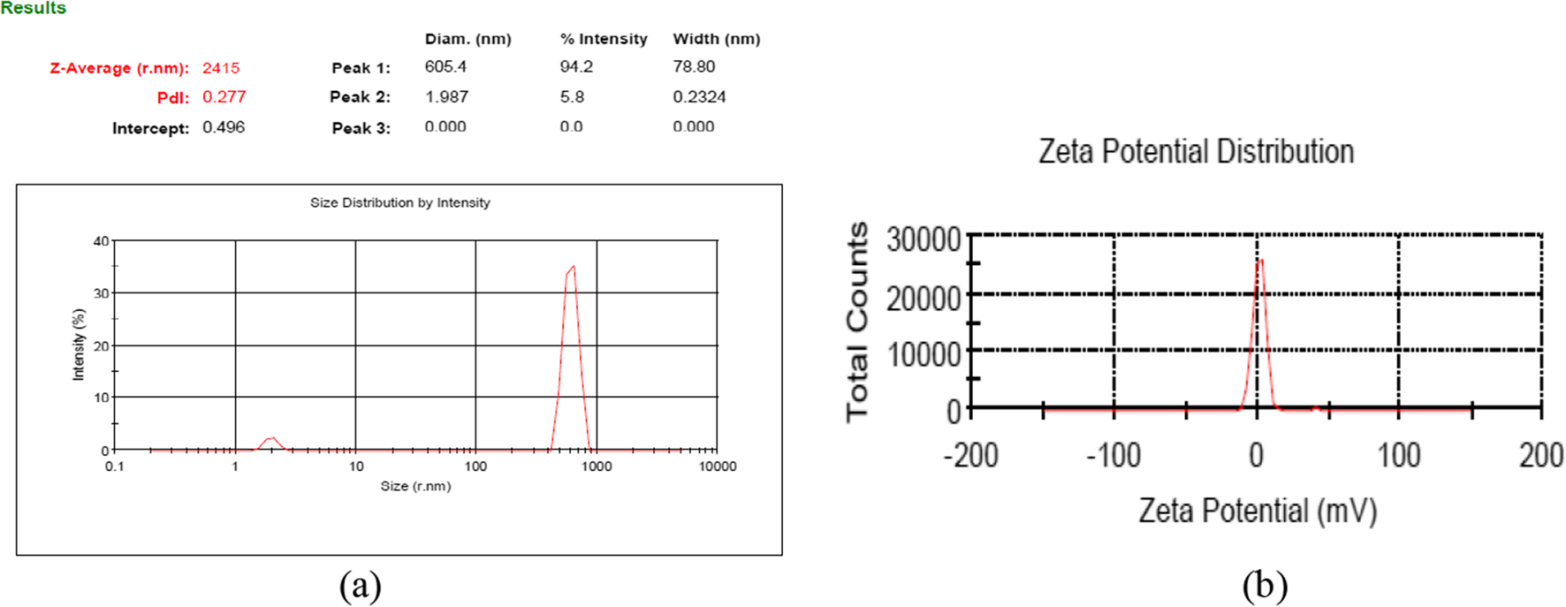
**a** Size distribution graph for the iron oxide nanoparticles, **b** Zeta potential diagram and the charge distribution of iron oxide particles

Dynamic light scattering (DLS) was utilized as a non-destructive method for determining the size of the particles. The light scattered by the particles in the suspension changes over time in a reversed relation with the particle diameter. The scattered light frequency by the fluid sample indicated the size of the particles in the range of 600–900 nm (Fig. 4a). This increase in diameter is higher than the size of the individual particles observed in the image by the electron microscope. This may be due to the polydispersity of the particles in the sample and/or the hydrodynamic radius of the particles (Yang et al. 2014; Williams et al. 2006; Kanagasubbulakshmi and Kadirvelu 2017; Cho et al. 2012; Wang et al. 2013).

The electrical surface charge of the nanoparticles was measured using the DLS device, indicating that the particles have colloidal stability with a zeta potential of 1.55 mV (Fig. 4b) (Berg et al. 2009; Bhattacharjee 2016; Arsalani et al. 2018; Malard et al. 2009; Talari et al. 2015).

Fourier-transform infrared spectroscopy (FTIR) was performed on the samples, and the analysis of the data based on the FTIR spectrum indicates that region 545 is related to Fe O and region 3470 is associated with the tensile vibration of the OH group present in Fe O (Fig. 3c) (Lim et al. 2013; Kanagasubbulakshmi and Kadirvelu 2017).

Raman spectroscopy, a molecular spectroscopy technique, has many similarities to infrared spectroscopy. It should be noted that the bonds with stronger peaks in the infrared spectrum will have weaker peaks in Raman spectroscopy. As a consequence, when evaluating the electronegativity of the bonds, the electron cloud was concentrated on the more electronegative atom. Therefore, when the Raman light hits, there is no observable polarization, resulting in a weak peak in Raman. In addition, since our nanoparticles have been synthesized from a biological source using a water solvent and Raman spectra do not interfere with water, the intensity of the observed peak is not related to the water molecules, and the least amount of polarizability reflects the vibrations. Hence, based on the considerations mentioned above, the results obtained from Raman spectroscopy, presented in Table 2 and Fig. 3d, indicate the presence of iron oxide NPs in the range (Slavov et al. 2010; Soler et al. 2012; Li et al. 2012; Yuvakkumar et al. 2014; Cheera et al. 2016; Demirezen et al. 2019a, b; Bachheti et al. 2019; Zolfigol and Yarie 2015; Salehiabar et al. 2018).

**Table 2:** The conditions and data for performing the Raman analysis

Date	14.06.2021 1	Acq. time (s)	8	Accumulations	1	Laser	785 nm_Edge
Spectro (cm^-1^)		Hole (µm)	500	Slit (µm)	100	Grating	1200 (750 nm)
Filter	100%	Objective	x10_VIS	ICS correction	Off	Range (cm^-1^)	
Date	14.06.2021 1	Acq. time (s)	8	Accumulations	1	Laser	785 nm_Edge
Spectro (cm^-1^)		Hole (µm)	500	Slit (µm)	100	Grating	1200 (750 nm)
Filter	100%	Objective	x10_VIS	ICS correction	Off	Range (cm^-1^)	

**Fig. 5:**
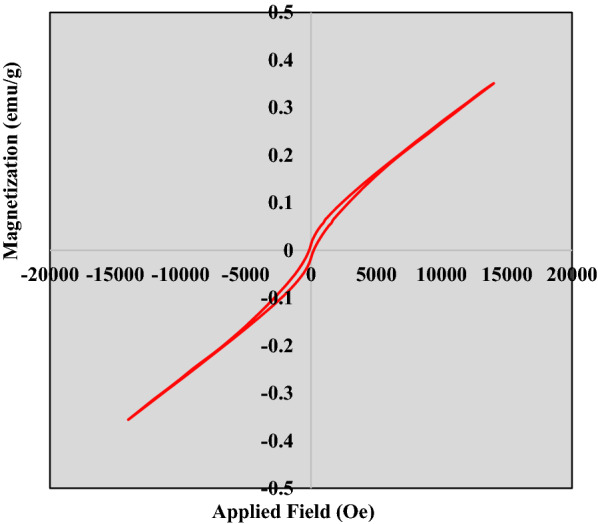
The VSM spectrum diagram

The magnetic behavior of the manufactured nanoparticle sample and the powder-like control was measured using VSM and by drawing the residual curve (Fig. 5). The results indicate that the manufactured sample has ferromagnetic behaviors with very low values of coercive force. Table 3 shows the values for magnetic parameters such as saturation magnetism, residual magnetism, and coercive force. The reduction in the saturation magnetism can be due to surface effects such as spin canting, the partial oxidation of the surface of the particles, shifting away from the stoichiometric state, and most importantly, the presence of the phases of impurity and the non-magnetic particles of these phases, affecting the value of this parameter (Yarie et al. 2016; Venkateswarlu et al. 2014; Elhamifar et al. 2018; Pérez-Beltrán et al. 2021).

**Table 3:** Values for magnetic parameters, including saturation magnetism, residual magnetism, and coercive force

Sample	Hc (Oe)	Ms (emu/g)	Mr (emu/g)
Control	25.21	0.11	0.0006
Fe ‌O	185.25	0.34	0.021

## Discussion

As noted earlier, metal nanoparticles are produced using various chemical and physical methods that impact their size, catalytic properties, and surface characteristics. Among various methods and sources used for the biosynthesis of metal nanoparticles, using bacteria as a source for producing metal NPs is especially significant. The current study uses distilled water as a non-polar solvent to avoid changes in the sample's polarity and a solvent without phase interference in all samples to dilute the samples in an attempt to increase the light passing through the sample. According to the Beer-Lambert law, the degree of light passing through a sample is expressed in percentage. Since the absorbance and passage of the light both lack a physical unit, we expressed light absorption as values in the wavelength using the unit of nanometers while considering light absorption as a significant reduction in the passing spectrum, indicating the presence of the specific material. Furthermore, since the height of the absorption peaks is directly related to the concentration of the material, the height of the absorption peak relative to the concentration of the sample indicated the accuracy of the nanoparticle synthesis. In addition, since the iron oxide nanoparticles had been synthesized in an aqueous environment, all analyses were performed on the colloidal solution of nanoparticles to ensure the accuracy of synthesis before investing the costs required for purification. Since the shift in the location of the peaks depends on factors such as the intensity of the laser shined on the sample and/or the size of the produced nanoparticles, we compared the results for our samples to the results obtained from the analyses of the controls selected for the current study as well as the results of previous studies. We made sure of the synthesis of iron oxide nanoparticles through these comparisons. The synthesized nanoparticles have a close hexagonal pack (HCP) structure. In this structure, the atoms of one of the alternating layers are localized to the gap between the atoms of previous layers, similar to an FCC structure. However, instead of a cubic structure, the structure is hexagonal. This is why the synthesized iron oxide shows rhombohedric grid characteristics, on the one hand, similar to a type of iron oxide called hematite ($$F{e}_{2}{O}_{3})$$ i.e., a brown to gray color, and on the other hand, it has a spherical shape with hexagonal grid characteristics as reported in the FESEM imaging. One reason for this phenomenon can be the difference in the grid parameter in different directions in the HCP structure. Another reason, according to the studies performed by Barlow (1883–1897), spherical particles are localized by maintaining the distance of twice the radius between the two centers of the spheres, except for a distance jump with a value of $$r$$ in the direction of the X-axis. In other words, the center of the sphere in the lower row is along the X-axis at the connection between the two spheres of the upper row. This causes the spheres in the new row to be closer to the spheres of the first row until all the spheres of the new row are in the location of the connection between the spheres in the upper row. Since the new spheres are in contact with the two spheres, their centers create an equilateral triangle with the centers of the surrounding spheres. The sides of this triangle have a length of$$2r$$. The configuration of these equilateral triangles results in the dense hexagonal structure of the synthesized iron oxide nanoparticles with the minimum possible distance. Consequently, the synthesized nanoparticles have a structure comparable to that of metals with a good density number (the ratio of c to a). Moreover, the crystal structure of metals has different levels of stability at different temperatures, which affects the characteristics of the synthesized metal nanoparticles and their applications. A week after the synthesis, we did not observe any changes in the grid characteristics of the nanoparticles, ensuring us that the produced nanoparticles are appropriate for pharmaceutical and industrial applications. As noted earlier, according to the results of FESEM imaging, the synthesized nanoparticles have a small size and an acceptable homogeneity based on the value of the PDI measure. Furthermore, the large size of the NPs relative to conventional chemotherapy agents or drugs based on biological macromolecules allows combining them with several supportive components, active pharmaceutical compounds, and/or surface correction. In addition, the smart selection of these components, depending on the application considered for the NPs synthesized in the current study, can significantly impact the resonance stability of the particles and facilitate their targeting and stimulus-based activation. The zeta potential analysis is used to determine the degree of repulsion/attraction or the electrostatic charges between the particles and a measure of stability in colloidal systems (Arias et al. 2018; Gentili 2020; Vangijzegem et al. 2019; Silva et al. 2019). The obtained results show the desired level of surface charge for the synthesized NPs and the paramagnetic property of these nanoparticles. Therefore, these NPs can be a good candidate for designing Janus particles as pharmaceutical nanocarriers capable of carrying hydrophilic and hydrophobic drugs and a good candidate for diagnosis purposes using the hyperthermia method. The solubility of these NPs in water can be considered a great solution for a group of anticancer medications with a low level of solubility in water. In addition, they can be placed inside the structure of Janus particles to create a structure with two functional parts, where one part is the magnetic iron NPs, and the other part can include various functional molecules (Estelrich et al. 2015; Willis et al. 2020). Therefore, the synthesized NPs can be used to produce nano dendrimers capable of carrying drugs for drug delivery to different tumor tissues with magnetic orientations under external magnetic fields (Gottimukkala et al. 2017; Venkateswarlu et al. 2014, 2014; Gottimukkala et al. 2017). Finally, by designing the experiment in a simple way and using the biological characteristics of Bacillus megatrium, we choose the incubation time in such a way that we produce smaller and more uniform nanoparticles with a different structure compared to the research of Sepideh Ghani and colleagues. With further investigations on the characteristics of the produced nanoparticles, several therapeutic capabilities of them are introduced to other researchers for practical and pre-clinical tests for use in therapy (Ghani et al. 2017).

## Data Availability

All data generated or analysed during this study are included in this published article [and its supplementary information files].
